# Overexpression of Partner of Numb Induces Asymmetric Distribution of the PI4P 5-Kinase Skittles in Mitotic Sensory Organ Precursor Cells in *Drosophila*

**DOI:** 10.1371/journal.pone.0003072

**Published:** 2008-08-27

**Authors:** Carolina N. L. R. Perdigoto, Louis Gervais, Erin Overstreet, Janice Fischer, Antoine Guichet, François Schweisguth

**Affiliations:** 1 Ecole Normale Supérieure, CNRS UMR8542, Paris, France; 2 Institut Jacques Monod, CNRS UMR, Paris, France; 3 Department of Molecular, Cell and Developmental Biology, University of Texas, Austin, Texas, United States of America; Centre de Regulacio Genomica, Spain

## Abstract

Unequal segregation of cell fate determinants at mitosis is a conserved mechanism whereby cell fate diversity can be generated during development. In *Drosophila*, each sensory organ precursor cell (SOP) divides asymmetrically to produce an anterior pIIb and a posterior pIIa cell. The Par6-aPKC complex localizes at the posterior pole of dividing SOPs and directs the actin-dependent localization of the cell fate determinants Numb, Partner of Numb (Pon) and Neuralized at the opposite pole. The plasma membrane lipid phosphatidylinositol (4,5)-bisphosphate (PIP2) regulates the plasma membrane localization and activity of various proteins, including several actin regulators, thereby modulating actin-based processes. Here, we have examined the distribution of PIP2 and of the PIP2-producing kinase Skittles (Sktl) in mitotic SOPs. Our analysis indicates that both Sktl and PIP2 reporters are uniformly distributed in mitotic SOPs. In the course of this study, we have observed that overexpression of full-length Pon or its localization domain (LD) fused to the Red Fluorescent Protein (RFP::Pon^LD^) results in asymmetric distribution of Sktl and PIP2 reporters in dividing SOPs. Our observation that Pon overexpression alters polar protein distribution is relevant because RFP::Pon^LD^ is often used as a polarity marker in dividing progenitors.

## Introduction

Ontogenesis of complex multicellular organisms involves the generation of different cell types. One mechanism by which cell fate diversity can be achieved is asymmetric cell division. During asymmetric cell division, progenitor cell polarity directs the orientation of the mitotic spindle and the asymmetric localization of cell fate regulators, thereby ensuring unequal segregation of these regulators [1], [2].

Each mechanosensory bristle located on the notum of *Drosophila* is composed of four different cells. These cells originate from a single sensory organ precursor cell (SOP) via a fixed lineage comprising four stereotyped asymmetric cell divisions [3]. The SOP first divides within the plane of the epithelium to generate a posterior pIIa cell and an anterior pIIb cell [4]. Two regulators of Notch receptor signaling, Numb and Neuralized (Neur), localize at the anterior cortex of dividing SOPs and segregate into the anterior daughter cell [5], [6]. Numb inhibits Notch whereas Neur positively regulates the signaling activity of the Notch ligand Delta. This therefore leads to Notch inhibition in the anterior cell that adopts the pIIb fate and Notch activation in the posterior cell that becomes pIIa.

By analogy with the role of atypical Protein Kinase C (aPKC) in neuroblasts [7], asymmetric localization of Numb and Neur at the anterior cortex of dividing SOPs is thought to depend on the kinase activity of aPKC, which localizes at the posterior pole. In brief, aPKC phosphorylates and inhibits Lethal (2) giant larvae (Lgl) at the posterior cortex, such that active nonphosphorylated Lgl protein is restricted to the anterior cortex where it promotes the cortical localization of Numb and Neur [7], [8]. Direct phosphorylation of Numb by aPKC may further contribute to restricting the localization of Numb to the anterior cortex [9]. Additionally, drug studies have shown that depolymerization of microfilaments prevents cortical localization of Numb and Neur in dividing SOPs, indicating that actin plays an essential role in their polar distribution [6], [10]. Lastly, anterior localization of Numb probably also involves its interaction with Partner of Numb (Pon). The Pon protein contains an N-terminal Numb-interacting domain, a central coiled-coil domain, and a C-terminal localization domain (LD) that is sufficient for its asymmetric localization in neuroblasts and SOPs. Pon colocalizes with Numb and is required, at least in neuroblasts, for its asymmetric localization [11], [12].

Phosphatidylinositol 4,5-bisphosphate (PIP2) is a phospholipid present at the inner leaflet of the plasma membrane that has a wide range of proposed functions [13]. PIP2 directly interacts with several actin regulators [14] as well as with proteins known to be involved in the process of asymmetric division, including Par3 [15], Neuralized [16] and Numb [17]. PIP2 is mostly produced by type I (PIP5KIs) phosphatidylinositol phosphate 5-kinases that use phosphatidylinositol 4-phosphate (PI(4)P) as a substrate [13]. The *Drosophila* genome encodes three predicted PIP5KIs: PIP5K59B, CG17471 and *skittles (sktl)* [18], [19]. Recently, the *sktl* gene has been shown to play a critical role in PIP2 synthesis in the oocyte (Gervais et al., unpublished). Interestingly, the *sktl* gene appears to be expressed in SOPs [18]. To begin studying the potential role of PIP2 in asymmetric cell division, we have examined here the localization of Sktl and PIP2 reporters in dividing SOPs. Our analysis indicates that PIP2 and Sktl are distributed at the cortex of dividing SOPs with no clear sign of planar asymmetry. However, in the course of these experiments, we have observed that an increased accumulation of Pon, which is known to accumulate at the anterior cortex in mitotic SOPs, resulted in the posterior localization of Sktl. We discuss here the practical implications and possible biological significance of this overexpression phenotype.

## Results

### Analysis of PIP2 distribution in mitotic SOPs

**Figure 1:**
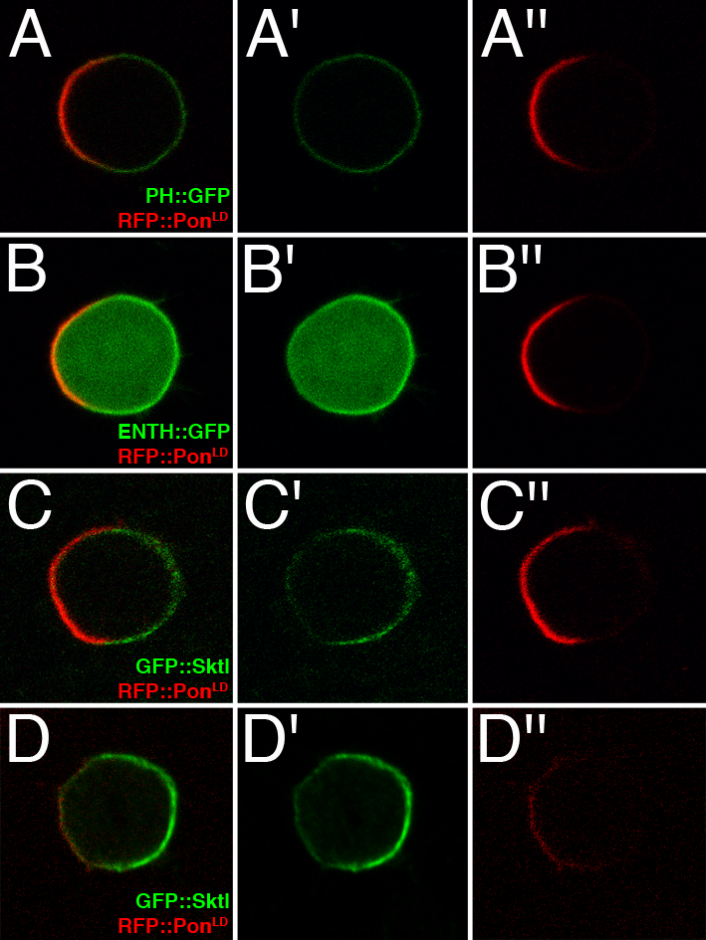
PIP2 reporters and GFP::Sktl localized asymmetrically in dividing SOPs expressing RFP::Pon^LD^. The distribution of PH::GFP (A,A′), ENTH::GFP (B,B′) and GFP::Sktl (C,C′) was examined by live imaging (A–C″) or by antibody staining (anti-GFP in green in D–D″) in dividing SOPs expressing RFP::Pon^LD^ (A″,B″,C″,D″). Both PIP2 reporters and GFP::Sktl (C; 83%; n = 18) localize at the posterior pole of dividing SOPs at prometaphase when co-expressed with RFP::Pon^LD^. All transgenes were expressed under the control of neur^P72^Gal4 Gal80^ts^. Anterior is on the right in this and all other figures.

The dynamics of PIP2 distribution can be followed in live cells using PIP2 reporters consisting of a PIP2-interacting domain fused to a fluorescent protein [20]. In this study, we have used the Pleckstrin Homology (PH) domain of the phospholipase Cδ1 fused to GFP (PH::GFP) [21] and the Epsin N-Terminal Homology domain of *Drosophila* Liquid facets (Lqf) [22] also fused to GFP (ENTH::GFP). These two PIP2 reporters were specifically expressed in SOPs using the UAS/GAL4 system [23] and their localization was monitored in living pupae. A fusion protein consisting of the Red Fluorescent Protein (RFP) fused to the C-terminal localization domain of Pon (RFP::Pon^LD^) [11], [24] was co-expressed with the PIP2 reporters to reveal SOP polarity. When co-expressed with RFP::Pon^LD^, PH::GFP was found to localize at the cell cortex and appeared to be slightly enriched at the posterior cortex (Fig. 1A–A″). ENTH::GFP was found to localize at higher levels at the posterior cortex (Fig. 1B–B″). ENTH::GFP was also found in the cytoplasm, perharps reflecting a lower affinity of the ENTH for PIP2 relative to the PH domain [25]. To test whether the posterior accumulation of these reporters correlated with the localization of the PIP2-producing kinase Sktl, we next examined the distribution of Sktl using a functional GFP::Sktl fusion protein (Gervais et al, unpublished). Similarly to PH::GFP and ENTH::GFP, GFP::Sktl cortical localization was concentrated at the posterior cortex, i.e. opposite to RFP::Pon^LD^ (Fig. 1C–D″; 83% n = 18).

**Figure 2:**
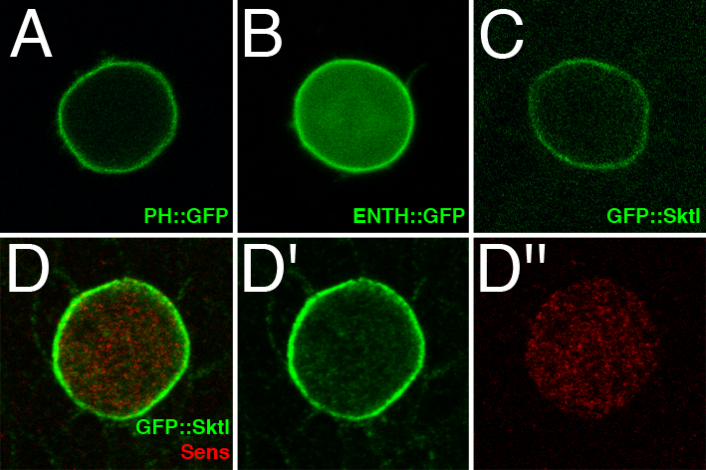
PIP2 Reporters and GFP::Sktl localized uniformly in SOPs in the absence of RFP::Pon^LD^. The distribution of PH::GFP (A), ENTH::GFP (B) and GFP::Sktl (C–D″) was examined by live imaging (A–C) or by antibody staining (anti-GFP in green and anti-Senseless in red in D–D″) in dividing SOPs at prometaphase. When RFP::Pon^LD^ was not co-expressed in SOPs, the PIP2 reporters and GFP::Sktl (panel C; 100%; n = 20) localized uniformly. All transgenes were expressed under the control of neur^P72^Gal4Gal80^ts^.

In the experiments described above, RFP::Pon^LD^ reporter was used simply as an internal control for asymmetry. However, we observed unexpectedly that when SOPs did not co-express RFP::Pon^LD^ the localization of PH::GFP, ENTH::GFP and GFP::Sktl was no longer polarized. PH::GFP (Fig. 2A) and ENTH::GFP (Fig. 2B) localized uniformly at the cell cortex. Similarly, GFP::Sktl localized uniformly at the cortex of dividing SOPs, both in living (Fig. 2C; 100% n = 20) and fixed cells (Fig. 2D–D′). Two conclusions can be drawn. First, PIP2 reporters and GFP::Sktl are distributed uniformly along the a–p polarity axis of wild-type SOPs at mitosis. Second, expression of RFP::Pon^LD^ has the ability to alter the distribution of PIP2 in dividing SOPs, possibly by altering the distribution of the PIP2-producing enzyme Sktl. Finally, the defective distribution of PIP2 seen upon RFP::Pon^LD^ expression may reveal a novel activity of Pon that is distinct from its known Numb-binding activity.

### Pon overexpression alters the distribution of Sktl

**Figure 3:**
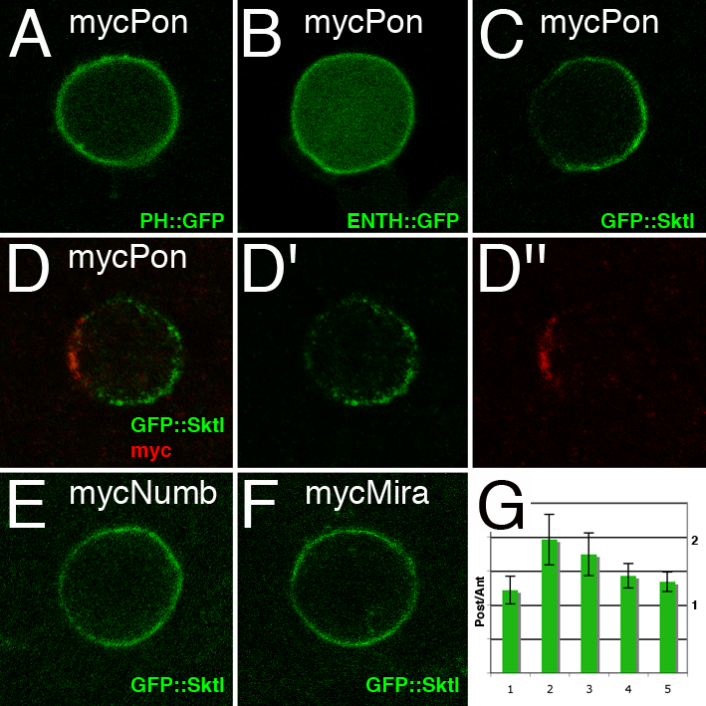
GFP::Sktl localized at the posterior pole upon Pon overexpression. Distribution of PH::GFP (A), ENTH::GFP (B) and GFP::Sktl (C–F) in SOPs co-expressing Myc-tagged versions of full-length Pon (mycPon; A–D″), Numb (mycNumb; E) or Miranda (mycMira; F). Pon, Numb and Mira localized at the anterior pole of dividing SOPs at prometaphase (anti-Myc in red in D″; data not shown). Overexpression of Pon did not detectably modify the localization of PH::GFP (A), ENTH::GFP (B) in living SOPs. However, Pon overexpression resulted in the posterior accumulation of GFP::Sktl in live SOPs (panel C; 56%; n = 9) as well as in fixed cells (D,D′). Overexpression of Numb (E; 100%; n = 14) or Mira (F; 100%; n = 9) did not change the distribution of GFP::Sktl in live SOPs. All transgenes were expressed under the control of neur^P72^Gal4Gal80^ts^. (G) Quantification of the relative GFP signal intensity measured at the posterior *vs* anterior cortex of dividing SOPs at metaphase. GFP::Sktl was expressed in combination with the following constructs: 1. none (as in Fig. 2C); 2. RFP::Pon^LD^ (as in Fig. 1C′); 3. mycPon (as in Fig. 3C); 4. mycNumb (as in Fig. 3E); 5. mycMira (as in Fig. 3F). Ratio values are (mean+/− standart deviation): 1.2 +/− 0.2; 2.0 +/− 0.4; 1.7 +/− 0.3; 1.4 +/− 0.2; 1.3 +/− 0.1. Expression of RFP::Pon^LD^ and mycPon, but not mycNumb nor mycMira, alters the distribution of GFP::Sktl in a statistically significant manner.

To test whether the activity seen with the C-terminal LD domain of Pon indeed reveals a novel activity of Pon, a tagged version of full-length Pon was overexpressed in dividing SOPs. While expression of Pon did not detectably alter the localization of PH::GFP (Fig. 3A) or ENTH::GFP (Fig. 3B), it resulted in the posterior localization of GFP::Sktl. This posterior accumulation was seen both in living (Fig. 3C; 56%, n = 9) and in fixed SOPs (Fig. 3D′; 55% n = 11). The difference in results with Pon and RFP::Pon^LD^ (compare Figs 2A,B and 3A,B) may be due to higher level of RFP::Pon^LD^ expression and/or activity. This difference suggests that the asymmetric distribution of PIP2, as monitored using the PH::GFP and ENTH::GFP reporters, does not necessarily correlate with those of GFP::Sktl and that GFP::Sktl may be more sensitive to the overexpression of Pon. Moreover, this effect of Pon appeared to be specific since overexpression of Numb or Miranda (Mira), two proteins that colocalize with Pon at the anterior cortex of dividing SOPs [11], [26] (our unpublished data), had no significant effect on the spatial localization of GFP::Sktl in dividing SOPs (Fig. 3E,F; Numb: 100%, n = 14; Mira: 100% n = 9; see quantification in Fig. 3G). We conclude that increasing the levels of Pon at the anterior pole of dividing SOPs prevents GFP::Sktl from localizing uniformly at the cell cortex.

**Figure 4:**
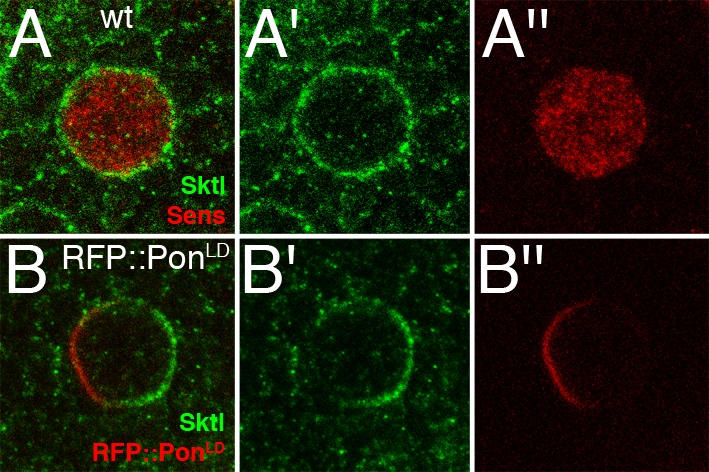
Endogenous Sktl localized uniformly in wild-type SOPs but accumulated at the posterior pole upon RFP::Pon^LD^ overexpression. Localization of endogenous Sktl (anti-Sktl in green) in wild-type (wt; A–A″) and RFP::Pon^LD^-expressing SOPs (B–B″). RFP::Pon^LD^ was expressed under the control of neur^P72^Gal4Gal80^ts^. Sktl localized uniformly in wt mitotic SOPs (A,A′; 100%; n = 15), which were identified by Sens staining (red in A,A″). When RFP::Pon^LD^ was overexpressed in SOPs, Sktl localized asymmetrically at the posterior pole (B,B′; 69%; n = 32).

We next examined whether expression of Pon can also perturb the localization of endogenous Sktl in SOPs. We therefore analyzed the distribution of Sktl in fixed nota using an anti-Sktl antibody that specifically recognize Sktl on fixed tissues (Gervais et al., unpublished). Sktl was detected at the apical cell cortex of all cells in the notum. We did not detect an increased accumulation of Sktl in SOPs. Sktl was found to accumulate uniformly at the cell cortex in dividing SOPs (Fig. 4A′; 100% n = 15). However, Sktl preferentially localized at the posterior cortex upon RFP::Pon^LD^ expression in SOPs (Fig. 4B′; 69% n = 32). Thus, overexpression of Pon modifies the spatial localization of endogenous Sktl during SOP asymmetric cell division (Fig. 4B′). As the mechanism whereby Pon and Sktl localize at the cell cortex are not known, how Pon may prevent GFP::Sktl accumulation at the anterior cortex is unclear.

## Discussion

We have shown that PIP2 and the PIP2-producing kinase Sktl are distributed at the cortex of dividing SOPs with no asymmetry within the plane of the epithelium. Whether PIP2 and Sktl regulate SOP polarization and/or polar localization of cell fate determinants in dividing SOPs remains to be further studied. Of note, we have not been able to analyze the phenotypes associated with a loss of *sktl* activity in the notum since *sktl* mutant clones fail to grow in wing imaginal discs (C.P., unpublished results).

We have also shown that overexpression of Pon can alter the spatial distribution of PIP2 binding proteins and of endogenous Sktl in dividing SOPs. This unexpected observation raises at least two questions and should also serve as a note of caution for studies using Pon as a marker of polarity.

Multiple mechanisms may explain how Pon overexpression alters the distribution of Sktl. One hypothesis is that both proteins compete for transport machinery components and/or cortical anchoring sites. Endogenous Pon would not affect the transport and/or anchoring of Sktl but overexpression of Pon could saturate the system. Also, the posterior pole accumulation of the PIP2-binding proteins PH::GFP and ENTH::GFP in Pon-overexpressing cells can be interpreted to suggest that overexpression of Pon results either in lower PIP2 concentration at the anterior pole or in higher concentration of competing PIP2-interacting proteins. It would also be interesting to examine whether the localization of the PIP2-producing kinase is itself regulated by PIP2 levels.

How may endogenous Pon regulate protein distribution at the cortex of asymmetrically dividing progenitors? A known function of Pon is to recruit Numb, most likely via direct protein-protein interaction [11], [12]. In this case, Pon plays a positive role by recruiting specific proteins at one pole of the dividing cell. However, our observation that Sktl accumulates at the posterior cortex when Pon is overexpressed raises the hypothesis that endogenous Pon may also normally exclude proteins from the membrane domain where it localizes.

Finally, our observation that Pon and RFP::Pon^LD^ alter the distribution of both PIP2 reporters and Sktl is relevant since both RFP::Pon^LD^ and GFP::Pon^LD^ have often been used to study asymmetric cell division of neural precursor cells in *Drosophila* [see: [8], [11], [26]] including detailed quantitative aspects of asymmetric protein localization [27]. As accumulation of free PIP2-binding sites at the posterior pole of RFP::Pon^LD^-expressing cells is likely to cause subtle deviations from the wild-type, our observation should serve as a note of caution when interpreting studies that use RFP::Pon^LD^ as a marker.

## Materials and Methods

The following transgenes were expressed using neur^P72^Gal4 [26] combined with a pTub-GAL80^ts^ transgene: ENTH::GFP [22], [28], PH::GFP [21], RFP::Pon^LD^ [24], Sktl::GFP (Gervais, in press), mycPon [11], mycMira [29], mycNumb [30].

Live GFP imaging was carried out as described in [26]. All images were acquired on SP2 and SP2AOBS confocal microscopes. Quantification of GFP signal intensity was performed using the plot profiling function of ImageJ 1.32.

Pupal nota were dissected from staged pupae and processed as previously described [3]. Primary antibodies were: rabbit anti-Sktl (Gervais et al. unpublished; 1∶500), guinea-pig anti-Senseless (Sens; gift from H. Bellen; 1∶3000), rabbit anti-GFP (Molecular Probes; 1∶1000) and mouse anti-Myc (9E10; DSHB; 1∶500). Cy3- and Cy5-coupled secondary antibodies were from Jackson's Laboratories and Alexa-488 coupled secondary antibodies were from Molecular Probes.
